# Patient-derived and artificial ascites have minor effects on MeT-5A mesothelial cells and do not facilitate ovarian cancer cell adhesion

**DOI:** 10.1371/journal.pone.0241500

**Published:** 2020-12-03

**Authors:** Manuela Estermann, Yen-Lin Huang, Dedy Septiadi, Danilo Ritz, Ching-Yeu Liang, Francis Jacob, Barbara Drasler, Alke Petri-Fink, Viola Heinzelmann-Schwarz, Barbara Rothen-Rutishauser

**Affiliations:** 1 Adolphe Merkle Institute, University of Fribourg, Fribourg, Switzerland; 2 Department of Biomedicine, University Hospital Basel and University of Basel, Basel, Switzerland; 3 Proteomics Core Facility, Biozentrum, University of Basel, Basel, Switzerland; 4 Department of Chemistry, University of Fribourg, Fribourg, Switzerland; University of Alabama at Birmingham, UNITED STATES

## Abstract

The presence of ascites in the peritoneal cavity leads to morphological and functional changes of the peritoneal mesothelial cell layer. Cells loose cell-cell interactions, rearrange their cytoskeleton, activate the production of fibronectin, and change their cell surface morphology in a proinflammatory environment. Moreover, ovarian cancer cell adhesion has been shown to be facilitated by these changes due to increased integrin- and CD44-mediated binding sites. In this study, the biological responsiveness of the human pleural mesothelial cell line MeT-5A to patient-derived and artificial ascites was studied *in vitro* and adhesion of ovarian cancer cells, i.e. SKOV-3 cells, investigated. Changes were mainly observed in cells exposed to artificial ascites containing higher cytokine concentrations than patient-derived ascites. Interestingly, reduced cell-cell interactions were already observed in untreated MeT-5A cells and effects on tight junction protein expression and permeability upon exposure to ascites were minor. Ascites induced upregulation of CDC42 effector protein 2 expression, which affects stress fiber formation, however significant F-actin reorganization was not observed. Moreover, fibronectin production remained unchanged. Analysis of mesothelial cell surface characteristics showed upregulated expression of intercellular adhesion molecule 1, slightly increased hyaluronic acid secretion and decreased microvillus expression upon exposure to ascites. Nevertheless, the observed changes were not sufficient to facilitate adhesion of SKOV-3 cells on MeT-5A cell layer. This study revealed that MeT-5A cells show a reduced biological responsiveness to the presence of ascites, in contrast to published studies on primary human peritoneal mesothelial cells.

## Introduction

Ovarian cancer is usually diagnosed at an advanced stage with metastases in the peritoneum and omentum resulting in low survival rates [1–3]. Therefore, a better understanding of disease progression within the omentum and efficient personalized drug testing is required to improve treatment options for ovarian cancer patients. The development of an omentum tissue barrier model would offer the possibility to investigate mechanisms of ovarian cancer cell adhesion and provide a platform for drug screening.

The surface of the omentum is covered with a layer of mesothelial cells [4]. They provide a slippery surface by secreting surfactant and glycosaminoglycans, mainly hyaluronic acid (HA), which is trapped between the microvilli on the apical side of the cells [4–6]. The cells also build tight cell layers to establish cell polarity and maintain a semi-permeable barrier [7]. The mesothelial cell layer represents the first point of interaction with malignant ascites, which accumulates within the peritoneal cavity. This fluid is excessively released from the lymphatic system in ovarian cancer patients [8,9] and contains cytokines, growth factors, extracellular matrix (ECM) proteins, and ovarian cancer cells [10,11]. It has been shown that exposure to ascites induced an epithelial-to-mesenchymal transition (EMT) in mesothelial cells *in vitro*, which leads in different phases to disruption of cell-cell interactions, cytoskeleton rearrangement, and increased fibronectin (FN1) production, among others [12–14]. Cell retraction, decreased stress fiber formation and upregulated FN1 secretion upon exposure to proinflammatory cytokines (i.e. tumor necrosis factor-α (TNF-α), interleukin-1β (IL-1β), transforming growth factor-β (TGF-β)) has been studied both *in vitro* and *in vivo* in mice [15–19]. Furthermore, cell surface changes, namely upregulated expression of intercellular adhesion molecule 1 (ICAM-1), vascular cell adhesion molecule 1 (VCAM-1), and HA, have been observed in primary human peritoneal mesothelial cells (pHPMC) exposed to cytokines (i.e. TNF-α, IL-1 and interleukin-6 (IL-6)) [20–24]. Additionally, microvillus expression of mesothelial cells exposed to ascites has been shown *in vivo* to be reduced in rats [16,25]. These alterations were shown to facilitate the adhesion of ovarian cancer cells mainly via (i) exposure of submesothelial ECM, where cancer cells preferably adhere, and via (ii) upregulated expression of ICAM-1/VCAM-1, HA, and FN1, providing integrin- and CD44-mediated ovarian cancer cell adhesion [11,17,18,22,26–29].

The mesothelial cell layer of the omentum model should respond to ascites as observed *in vivo*. Previous studies performed experiments with isolated pHPMC to investigate effects of ascites on mesothelial cells and cancer cell adhesion. The scope of this study was to evaluate the biological responsiveness of the non-cancerous pleural mesothelial and commercially available cell line MeT-5A to patient-derived and artificial ascites and its possible application for ovarian cancer adhesion studies. We assessed (i) disruption of cell-cell interactions, (ii) cytoskeleton rearrangement, (iii) FN1 production, and (iv) cell surface changes (i.e. ICAM-1, VCAM-1, HA and microvillus expression) of MeT-5A cells upon exposure to ascites. The impact of ascites on interactions between ovarian cancer and mesothelial cells was investigated with regard to SKOV-3 cell adhesion on the MeT-5A cell layer. In addition, the effects of ascites on cell viability, proliferation, apoptosis, and the cellular response to the proinflammatory environment were studied. Here, we provide evidence that MeT-5A cells show reduced biological responsiveness when exposed to ascites in comparison to results presented in previously published studies using pHPMC [12]. These limitations should be considered when MeT-5A cells are used for the investigation of the adhesion of ovarian cancer cells.

## Material and methods

### Cell culture

MeT-5A: The human mesothelial cell line MeT-5A was purchased from American Type Culture Collection (ATCC^®^ CRL-9444^™^, LGC Standards, Germany) and cultured in Medium 199 (M199, 31150022, Gibco, Thermo Fisher Scientific, Switzerland) supplemented with 10% (v/v) fetal bovine serum (FBS, Gibco, Thermo Fisher Scientific, Switzerland), 1% (v/v) penicillin/streptomycin (P/S, Gibco, Thermo Fisher Scientific, Switzerland), 20 mM HEPES (H0887, Sigma-Aldrich, Switzerland), 400 nM hydrocortisone (H0888, Sigma-Aldrich, Switzerland), 870 nM insulin (I0516, Sigma-Aldrich, Switzerland), 0.3869 mg/L sterile filtered selenious acid (211176, Sigma-Aldrich, Switzerland), 3.3 nM human epidermal growth factor (EGF, 130-097-749, Miltenyi Biotec Inc., Germany) and 1:1000 Trace Elements B (25-022-CI, Corning™, Thermo Fisher Scientific, Switzerland). Cell lines were authenticated using short tandem repeat (STR) profiling and regularly tested for the absence of mycoplasma. Cells were subcultured twice per week according to the manufacturer’s instructions and used for experiments from passage 3 to 25.

SKOV-3: The human ovarian cancer cell line SKOV-3 (ATCC^®^ HTB-77^™^, LGC Standards, Germany) was lentivirally transduced with pUltra (#24129, addgene, USA) for constitutive expression of enhanced green fluorescent protein (EGFP). Briefly, HEK293T cells (ATCC^®^ CRL-3216^™^, LGC Standards, Germany) were transfected with 4 μg of pUltra, 2 μg of pMD2.G (#12259, addgene, USA), and 2 μg of pCMVR8.74 (#22036, addgene, USA) using 24 μL of jetPEI reagent in 1 mL of 150 mM NaCl solution (Polyplus-transfection, Chemie Brunschwig AG, Switzerland). Medium was changed 24 h after transfection. Virus supernatant was harvested after 48 h and filtered with a 0.45 μm polyvinylidene fluoride filter (Millipore, Switzerland). Subsequent lentivirus-containing medium was used to transduce SKOV-3 cells. SKOV-3 cells expressing EGFP were sorted by a BD FACSAria™ Cell Sorter (BD Bioscience, Switzerland) and further cultured in Roswell Park Memorial Institute 1640 Medium (RPMI-1640, 42401–018, Gibco, Thermo Fisher Scientific, Switzerland) supplemented with 10% (v/v) FBS, 1% (v/v) L-glutamine and 1% (v/v) P/S. Cell lines were authenticated using STR profiling and regularly tested for the absence of mycoplasma. Subculturing was performed twice a week according to the manufacturer’s instructions and used for experiments from passage 3 to 25.

### Artificial and patient-derived ascites

Artificial ascites (ArA) were prepared by mixing 10 ng/mL TNF-α (Ref. 11343015, ImmunoTools, Germany), 250 pg/mL IL-1β (Ref. DY201, R&D Systems, Switzerland), 1 ng/mL IL-6 (Ref. DY206, R&D Systems, Switzerland) and 10 ng/mL TGF-β1 (Ref. 11343160, ImmunoTools, Germany) in complete M199 (cM199). The cytokine concentrations were chosen based on reported concentrations, which induced morphological and functional alterations in pHPMC *in vitro* [15,18,22,24,30,31]. Patient-derived ascites were collected from the abdominal cavity of patients suffering from ovarian or peritoneal cancer at the University Hospital in Basel. The ethical committee Nordwest- and Zentralschweiz, Switzerland (EKNZ, BASEC ID: 2017–01900) specifically approved this study. The informed written consent was obtained from all patients. Ascites were collected from three different donors diagnosed with FIGO stage III high-grade serous ovarian cancer (OvC3), FIGO stage IV high-grade serous ovarian cancer (OvC4), and FIGO stage IV high-grade serous peritoneal cancer (PeC) (S1 Table). Ascites samples were sterile filtered (4652, Pall, Germany) and stored at –80°C.

### Cytokine concentration of patient-derived ascites

The TNF-α, IL-1β, IL-6, and TGF-β1 concentrations of patient-derived ascites were assessed via enzyme-linked immunosorbent assay (ELISA) using the commercially available human TNF-α DuoSet^®^ ELISA (Ref. DY210, R&D Systems, Switzerland), human IL-1β/IL-1F2 DuoSet^®^ ELISA (Ref. DY201, R&D Systems, Switzerland), human IL-6 DuoSet^®^ (Ref. DY206, R&D Systems, Switzerland), and human TGF-β1 DuoSet^®^ ELISA (Ref. DY240, R&D Systems, Switzerland). So that the readings were within the detection limit, samples were diluted in reagent diluent. The following dilutions were used for the tested cytokines: undiluted for TNF-α and IL-1β, 1:8 (OvC3 and OvC4), and 1:40 (PeC) dilution for IL-6, and 1:10 for TGF-β1. Assays were performed according to the manufacturer’s instructions and measurements were conducted in triplicate.

### Sample preparation

A total of 1∙10^5^ MeT-5A cells were seeded in 500 μL complete M199 (cM199) on 12-well Falcon^®^ cell culture inserts (n = 3, Ref. 353091, BD Biosciences, Switzerland) with a pore size of 3 μm and a cell growth area of 0.9 cm^2^. Cells were cultured for three days to obtain a confluent layer and then exposed for two days to either cM199 or ascites to simulate healthy and diseased environments, respectively [15]. Cell culture medium (i.e. cM199) or ascites (500 μL) were added to the upper chamber of the inserts and 1.5 mL of cM199 only was added to the lower part. After two days of exposure, the supernatant was collected from the lower and upper chamber and stored at –80°C until further use. Cell samples were either fixed for laser scanning microscope (LSM) and scanning electron microscope (SEM) imaging or immediately used for permeability testing. All experiments were repeated three times with different cell passage numbers. In parallel, ascites control samples were prepared by mixing 500 μL ascites with 1.5 mL of cM199. Samples were stored for two days in the incubator and then kept at –80°C until further analyses. Inserts used to evaluate SKOV-3 cell adhesion were coated with 0.03% bovine collagen type I (CB-40231, Corning™, Thermo Fisher Scientific, Switzerland) and 0.01% human fibronectin (CB-40008, Corning™, Thermo Fisher Scientific, Switzerland) in 0.1% bovine serum albumin (BSA, Sigma-Aldrich, Switzerland) in 1X phosphate-buffered saline (PBS, Gibco, Thermo Fisher Scientific, Switzerland). MeT-5A cells were cultured on inserts for three days as described above and exposed to ascites for two days. SKOV-3 cells (2∙10^4^) were then resuspended in 500 μL cM199 and allowed to adhere for one day.

### Sample preparation for laser scanning microscopy

Samples were washed with PBS, fixed with 4% (w/v) paraformaldehyde in PBS (PFA, Sigma-Aldrich, Switzerland) for 15 min and then immersed in 0.1 M Glycine (Thermo Fisher Scientific, Switzerland) for another 15 min. Next, samples were immersed with the primary antibody, sheep polyclonal hyaluronic acid (ab53842, Abcam, United Kingdom, at 50 μg/mL) for 2 h at room temperature. Samples were then washed thoroughly, and the secondary antibody, donkey polyclonal anti-sheep Alexa Fluor^®^ 488 (ab150177, Abcam, United Kingdom, at 20 μg/mL), was added. Additionally, F-actin and nuclei were stained using rhodamine-phalloidin (R415, Thermo Fisher Scientific, Switzerland) and DAPI (4’,6-diamidino-2-phenylindole, 10236276001, Sigma-Aldrich, Switzerland), respectively. The samples were then washed, mounted on microscope slides using Glycergel^®^ (Dako, Denmark), and stored in the dark at 4°C. Images (1024 x 1024 pixels) were acquired using LSM (LSM 710 meta, Zeiss, Germany) and processed using Fiji (ImageJ, NIH, US).

### Sample preparation for scanning electron microscopy

Samples were fixed with a 1:1 mixture of 4% (w/v) PFA in PBS and 5% (v/v) glutaraldehyde (GA, Sigma-Aldrich, Switzerland) in 0.03 M K_2_HPO_4_ / KH_2_PO_4_ overnight at 5°C. Next, samples were dehydrated using 20%, 40%, 60%, 80% and 100% (v/v) absolute ethanol (AnalaR NORMAPUR^®^, VWR Chemicals, Switzerland) in deionized water each for 5 min, immersed in hexamethyldisilazane (HMDS, Sigma-Aldrich, Switzerland) for 15 min and left to dry overnight in a chemical fume hood. Samples were sputter-coated with a 3 nm gold layer and imaged using SEM (Tescan Mira3 LM FE, Germany).

### Lactate dehydrogenase assay

The supernatants of the upper and lower well chamber of cells exposed to ascites and of untreated cells were mixed in a ratio of 1:3 (v/v). Ascites control samples (patient-derived and artificial ascites diluted 1:3 in medium) and medium were used to evaluate the background absorbance. As a positive control, cells were exposed to 0.2% (v/v) Triton™ X-100 (Sigma-Aldrich, Switzerland) for 30 min. Lactate dehydrogenase (LDH) assay (11644793001, Roche, Sigma-Aldrich, Switzerland) was performed according to the manufacturer’s instructions.

### Fluorescein isothiocyanate-dextran assay

A permeability assay was performed as described elsewhere [32]. Briefly, exposed and untreated cells were immersed in Hank’s Balanced Salt Solution without salts (HBSS, H6648, Sigma-Aldrich, Switzerland), whereas 5 mM ethylenediaminetetraacetic acid (EDTA, Sigma-Aldrich, Switzerland) in HBSS was added to the upper chamber of positive controls. Samples were kept at 37°C for 1 h and HBSS and EDTA were aspirated thereafter. Then, 600 μL and 250 μL HBSS was added to the lower and upper chambers, respectively, of untreated and exposed cells and blank samples. Blank samples were inserts without cells and used as a background control. Next, 250 μL of 10 mM EDTA in HBSS was added to the upper chamber of the positive control. To assess the permeability through the cell layer, 250 μL of 4 mg/mL 70 kDa fluorescein isothiocyanate-dextran (FITC-dextran, 46945, Sigma-Aldrich, Switzerland) in HBSS was added to the upper chamber of all samples, resulting in a final concentration of 2 mg/mL FITC-dextran. Samples were incubated in the dark at 37°C for 1 h. The supernatant of the lower chamber was collected and kept in the dark upon measurements. Measurements were performed in triplicate using a microplate reader (Tristar LB 941, Berthold Technologies, Germany) and a filter at excitation and emission wavelength (λ_ex_ / λ_em_) of 485 / 535 nm. Three replicates per experiment were analyzed, resulting in nine data points. Results were normalized first to the median of all nine blank inserts and then to untreated cells.

### Gray-level co-occurrence matrix and principal component analysis

Alterations in F-actin organization were evaluated using LSM combined with image processing based on the gray-level co-occurrence matrix (GLCM) technique [33]. Briefly, nine LSM images (1024 × 1024 pixels) per condition were processed using Fiji. Four smaller image regions with the size of 500 × 500 pixels were randomly chosen using the duplicate toolbox available in Fiji (total data consisted of 36 images). The images were then split into their gray-level channels: nuclei and F-actins. For the latter, five important texture parameters, namely angular second moment, contrast, correlation, inverse difference moment, and entropy were extracted using the GLCM toolbox available in Fiji. In addition, the average F-actin intensity was determined using the histogram function in Fiji, while the total number of nuclei that corresponds to the number of cells in an image was counted manually. All datasets (texture parameters) were evaluated using multivariate analysis based on principal component analysis (PCA) [34] in Matlab (MathWorks, USA) to determine principal component (PC) scores. PCA is a data reduction technique that is used to reduce the dimensionality of such parameters, allowing plotting of the complex datasets in a simple two-dimensional (2D) plot, thereby increasing interpretability while retaining most of the variation in the data set [35]. Two PC scores (i.e. PC1 and PC2) possessing the highest variance in the data set are plotted as a 2D graph.

### Number of microvilli

Microvillus expression of mesothelial cells was assessed using SEM. The number of microvilli per image (1024 × 1144 pixels, a total of 18 images (six images per experiment and treatment)) were counted individually and the number of microvilli per μm^2^ was normalized to untreated cells.

### Interleukin-6 and hyaluronic acid concentration

IL-6 and HA concentration secreted by mesothelial cells was assessed using the commercially available human IL-6 DuoSet^®^ and human Hyaluronan Quantikine ELISA kit (Ref. DY206; Ref. DHYAL0, R&D Systems, Switzerland). The supernatant of cells exposed to ascites and untreated cells was assessed. The supernatants of the upper and lower chambers were mixed in a ratio of 1:3. Ascites control samples were also evaluated to analyze the background IL-6 and HA concentrations. The following dilutions were used: undiluted (OvC3 and OvC4) and 1:20 (PeC, ArA) for IL-6. Samples for HA ELISA were diluted 1:500. The assay was performed according to the manufacturer’s instructions and measurements were conducted in triplicate. The IL-6 and HA concentrations of the ascites control samples were subtracted from the measured values to visualize IL-6 and HA secreted by cells. Three replicates per experiment were analyzed, resulting in nine data points.

### Proteomics

Proteomics was performed to identify differences in the protein expression of MeT-5A cells exposed to ascites compared to untreated cells. Briefly, exposed and untreated cells were washed with cold PBS, scraped from inserts, and centrifuged and washed with cold PBS three times. PBS was removed and cell pellets were stored at –80°C. Next, cells were lysed and the protein concentration analyzed using the Pierce™ bicinchoninic acid (BCA) assay kit (23225, Thermo Fisher Scientific, Switzerland). Protein lysates were alkylated and digested with trypsin overnight at 37°C. Digested peptides were cleaned using an in-StageTip cartridge (ProOmics, Germany), resuspended in 0.1% formic acid and subjected to liquid chromatography-mass spectrometry/mass spectrometry (LC-MS/MS) analysis using an Orbitrap Fusion Lumos Tribrid Mass Spectrometer fitted with an EASY-nLC 1200 (Thermo Fisher Scientific, Switzerland). Peptides were resolved using a RP-HPLC column (75 μm × 36 cm) packed in-house with C18 resin (ReproSil-Pur C18–AQ, 1.9 μm resin; Dr. Maisch GmbH, Germany) at a flow rate of 200 nL/min. Raw files were imported into Progenesis QI software (v2.0, Nonlinear Dynamics Limited, United Kingdom) to extract peptide precursor ion intensities across all samples and were run against the human database containing 20,416 protein sequences. The relative quantitative data obtained were normalized and statistically analyzed using the in-house script Safe Quant (PMID:27345528). The quantitative analysis resulting from label-free quantification was processed using the SafeQuant R package v.2.3.2. (https://github.com/eahrne/SafeQuant/) to obtain peptide relative abundances. The summarized peptide expression values were used for statistical testing of differentially abundant peptides between conditions (S1 File).

### SKOV-3 cells adhesion

The staining of fixed samples was performed as described above using rhodamine-phalloidin and DAPI. Samples were imaged using LSM and z-stacks (1024 × 1024 pixels) of 12 areas per condition were captured. To evaluate the area of attached SKOV-3 cells, z-projection analysis with maximal intensity was applied and the percentage area was evaluated using Fiji.

### Statistical analysis

Normal distribution of data was analyzed using the Shapiro-Wilk normality test (Origin 2016, OriginLab Corporation, USA). One-tailed *t*-tests or one-way ANOVA with Sidak-Holm multiple comparisons were performed for normally distributed data (Prism 6, GraphPad, USA). For non-parametric data Kruskal-Wallis test on ranks and Dunn’s multiple comparisons versus control was performed (Prism 6). A *p*-value of less than 0.05 was considered statistically significant. Data were normalized to untreated cells of each experiment and outliers were identified (ROUT test, Prism 6) and excluded from further analysis. Data were presented as Min / Max boxplots. A value of 1 indicates values equal to those of untreated cells, whereas values below and above represent lower or higher values, respectively. For proteomics data, empirical Bayes moderated *t*-tests were applied. A *p*-value of less than 0.01 was considered statistically significant.

## Results and discussion

### Cytokine concentration in patient-derived and artificial ascites

The TNF-α, IL-1β, IL-6, and TGF-β1 concentrations in patient-derived ascites were evaluated, as these cytokines were shown to be key factors in inducing structural and functional alterations in mesothelial cells [15]. Moreover, the secretion of these cytokines (TNF-α, IL-6, and TGF-β) weaken the host antitumor immunity and allow the cancer cells to escape the immune system, which has been activated through the proinflammatory process of cancer progression and metastasis [36]. The determined cytokine concentrations of OvC3 and OvC4 (Table 1) were highly comparable and in the range of previously reported values for advanced ovarian cancer ascites [36–41], thus are representative for this cancer stage. PeC, which displays greater involvement in extraovarian sites with microscopically tumor-free ovaries, showed slightly higher TNF-α, similar IL-1β, and significantly increased IL-6 and TGF-β1 concentrations when compared to OvC3 and OvC4 (Table 1). This donor was included in the study as primary peritoneal cancer shares many clinical and morphological similarities with high-grade serous ovarian cancer and allowed to investigate the effects of ascites containing higher cytokine concentrations [42]. Primary peritoneal cancer is a rare and poorly investigated disease [42] and a precise cytokine profiling of its ascites was not yet reported. The cytokine concentrations are, however, within the range of advanced ovarian cancer ascites as described elsewhere [36–40]. We expect to have comparable conditions, as the ascites of all donors were collected at primary diagnosis (chemo-naïve) from patients with high-grade advanced stage serous adenocarcinoma showing high levels of CA125 and the pathologically confirmed presence of malignant cells (S1 Table). The measured cytokine concentrations of the three donors were compared to reported values, which previously showed to cause morphological and functional alterations in pHPMC *in vitro* (S1 Fig) [15,18,22,24,30,31]. It was shown that a minimum concentration of 100 pg/mL TNF-α or IL-1β, or 5 ng/mL TGF-β, is required to induce cell retraction in pHPMC *in vitro* [15,43]. FN1 production was significantly increased when cells were exposed to 250 pg/mL TGF-β and an increase of FN1 mRNA was observed when cells were exposed to 100 pg/mL IL-1β [18,19]. Furthermore, a concentration of 5 ng/mL TNF-α or IL-6 has been found to increase ICAM-1 and 10 ng/mL TNF-α induced VCAM-1 expression [22,30]. HA secretion was increased when pHPMC were exposed to 1 ng/mL TNF-α, 10 pg/mL IL-1β or 0.1 μg/mL TGF-β [44]. Elsewhere, it was reported that 10 ng/mL TNF-α, IL-6 or TGF-β doubled HA secretion, whereas the same concentration of IL-1β significantly increased HA secretion by a factor of eight [24]. The cytokine concentrations, which caused alterations in pHPMC *in vitro* are, however, predominantly at the upper end of the reported cytokine concentration ranges of patient-derived ascites. We presume that a higher cytokine concentration is required to induce changes in *in vitro* cell culture conditions compared to the *in vivo* situation, where the mesothelial cells are exposed to cytokines over a longer time. Therefore, the cytokine concentrations of ArA were chosen based on the reported values from the pHPMC *in vitro* studies. The use of both patient-derived and artificial ascites allows us to investigate the responsiveness of MeT-5A cells in an environment more similar to the *in vivo* situation, but also to indirectly compare the cell line with primary cells from previous studies.

**Table 1:** Cytokine concentration of patient-derived (OvC3, OvC4, PeC) and artificial ascites (ArA).

Ascites	TNF-α [pg/mL]	IL-1β [pg/mL]	IL-6 [pg/mL]	TGF-β1 [pg/mL]
OvC3	34.1	18.6	1035.4	2274.7
OvC4	14.1	0	833.7	1823.6
PeC	95.6	9.9	6322.7	7073.9
ArA	10’000	250	1000	10’000

### MeT-5A cell viability is not affected by exposure to ascites

**Fig 1:**
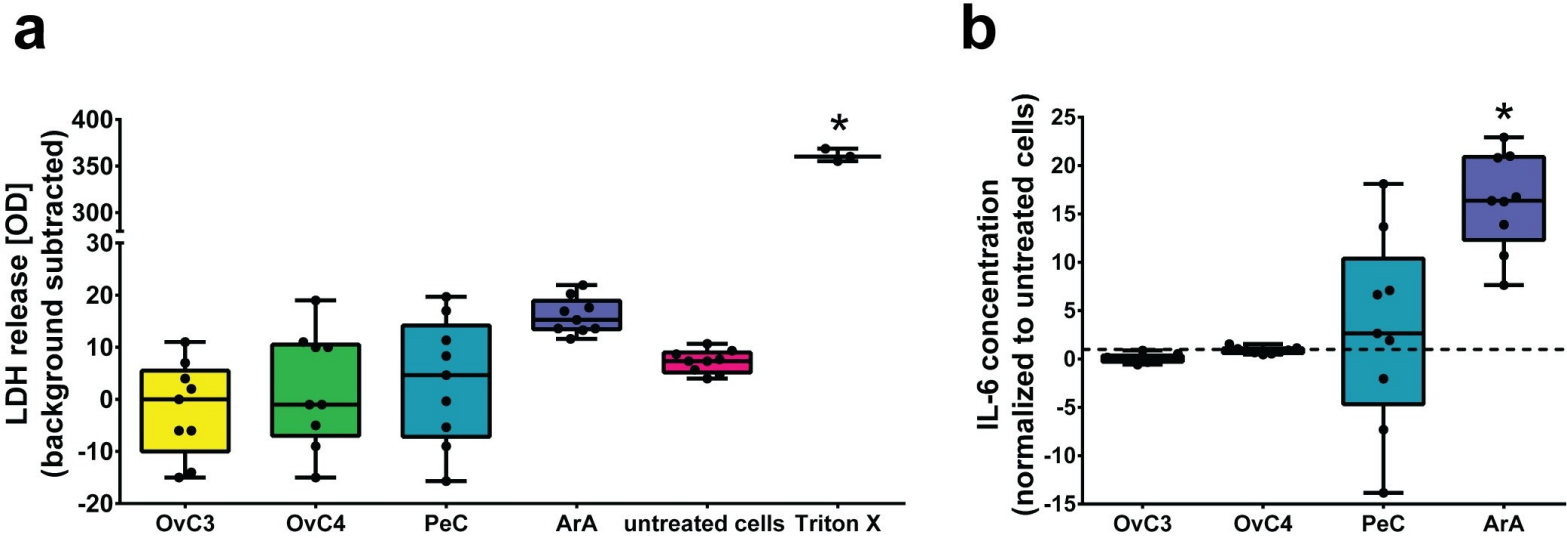
Cell viability and IL-6 secretion of ascites-exposed MeT-5A cells. a) LDH absorbance of cells exposed to ascites, untreated cells, and cells exposed to the positive control Triton™ X-100 (background subtracted); * *p*-value < 0.0001. b) IL-6 secretion of cells exposed to ascites, normalized to untreated samples (dotted lines); * *p*-value < 0.05. Data are presented as median ± Min/Max, n = 3.

The cell viability of ascites-exposed and untreated MeT-5A cells was evaluated using the LDH assay. As patient-derived ascites can contain basal concentrations of LDH [45], thereby falsifying the results, the absorbance of the ascites control samples and medium (background) were additionally evaluated. These values were then subtracted from the measured values to exclusively obtain the contribution of the MeT-5A cells. Cells treated with OvC3, OvC4, PeC, and ArA showed a median absorbance of 0, –1, 5, and 15, respectively. Untreated cells showed an absorbance of 7 (Fig 1A). Cells exposed to the positive control Triton™ X-100, which causes cell membrane disruption leading to cell death, consequently releasing LDH [46], showed a significantly higher median absorbance of 360, which was 50-fold higher than untreated cells (*p*-value < 0.0001, one-way ANOVA). Neither patient-derived nor artificial ascites affected cell viability.

### Artificial ascites induced anti-inflammatory response in MeT-5A cells

The inflammatory response of MeT-5A cells upon exposure to ascites was evaluated by measuring the IL-6 secretion and analyzing the intracellular protein expression using ELISA and proteomics, respectively. ArA and PeC led to 16.37-fold (Kruskal-Wallis test, *p*-value < 0.05) and 2.66-fold (*p*-value > 0.05) increased IL-6 secretions, respectively (Fig 1B). Cells exposed to OvC3 and OvC4, however, showed even lower concentrations than untreated cells (0.06- and 0.89-fold, *p*-value > 0.05). The exposure of mesothelial cells to proinflammatory cytokines in the form of TNF-α and IL-1β converts the cells into an “activated” phenotype [15] and can lead to an upregulated IL-6 secretion [20]. It was suggested that this upregulation provides a negative feedback signal for the production of IL-1 and TNF-α from macrophages and contributes to the control of the inflammation [31]. This observation was verified by analyzing the expression of disintegrin and metalloproteinase domain-containing protein 17 (ADAM17), which is responsible for the proteolytic release of membrane-bound TNF-α and IL-6, among others [47]. Cells exposed to ArA showed a statistically significant upregulation, while cells exposed to OvC3, OvC4, and PeC remained unchanged (Table 2, empirical Bayes moderated *t*-tests, *p*-value: < 0.001). Stimulation of cells with TNF-α upregulates the expression of genes involved in the control of tissue inflammation and triggers apoptosis-related cell death via the nuclear factor kappa B (NF-κB), mitogen-activated protein kinase (MAPK)/C-Jun, and caspase/apoptotic pathways [48,49]. Interleukin-1 receptor-associated kinase 1 (IRAK1) and lymphotoxin β receptor (LTβR) are both involved in the regulation of the inflammation. IRAK1 plays a key role in the signaling cascades of the toll-like and IL-1 receptors, while LTβR acts as a receptor for lymphotoxin expressed by immune cells and activates the NF-κB pathway [50–52]. Both proteins were significantly downregulated for cells exposed to ArA (Table 2, *p*-value: < 0.001), while cells exposed to OvC3, OvC4, and PeC remained unchanged. A downregulation of IRAK1 and LTβR inhibited both the TNF-α or IL-1β and lymphotoxin induced NF-κB activation [51,53], which has an anti-inflammatory effect and might be a mechanism to protect cells from cytokine-induced cell damage [52,54]. Based on these results we assume that MeT-5A cells attempt to control the inflammation by providing a negative feedback signal for cytokine release of macrophages [31] and inducing an anti-inflammatory effect to protect cells from cytokine-induced cell damage. Interestingly, only MeT-5A cells exposed to ArA induced this anti-inflammatory effect, which may be an indication that higher cytokine concentrations are required to induce effects in MeT-5A cells.

**Table 2:** Binary logarithm of the ratio of treated to untreated cells.

	OvC3	OvC4	PeC	ArA
Cellular response to a proinflammatory environment				
ADAM17	ns	ns	ns	**7.7**
IRAK1	ns	ns	ns	**-3**
LTBR	ns	ns	ns	**-5**
Cell proliferation and apoptosis				
CRLF3	**4.9**	**4.8**	**5.3**	**5.1**
CCNB2	**-2.4**	**-2**	**-1.9**	ns
TRF2	ns	ns	ns	**3.8**
BAD	**-2.2**	ns	ns	**-2.4**
Cell morphology and functionality related to cancer cell adhesion				
ZO-1	ns	ns	ns	ns
ZO-2	ns	ns	ns	ns
CDC42EP2	**1.8**	ns	ns	**2.4**
ACTA2	ns	ns	ns	**-3.6**
ICAM-1	ns	ns	ns	**1.0**
VCAM-1	ns	ns	ns	ns
FN1	ns	ns	ns	ns

Statistically significant values (*p*-value < 0.01) are shown, ns: non significant.

### Effects of artificial ascites on cell cycle arrest and survival in MeT-5A cells

Ascites-induced changes in cell behavior, e.g. cell proliferation and apoptosis, were evaluated by analyzing specific intracellular protein expression. We observed that the protein expression of cytokine receptor-like factor (CRLF3), G2/mitotic-specific cyclin-B2 (CCNB2), telomeric repeat-binding factor 2 (TRF2), and bcl-2 associated agonist of cell death (BAD) was significantly changed when compared to untreated cells. The expression of CRLF3 was upregulated under all applied conditions (Table 2, empirical Bayes moderated *t*-tests, *p*-value: < 0.001 (OvC3), 0.0023 (OvC4), 0.0043 (PeC), and 0.0011 (ArA)) and CCNB2 was found to be downregulated in cells exposed to OvC3, OvC4, and PeC (Table 2, *p*-value: 0.0034, 0.004, and 0.002). TRF2 was statistically significant upregulated in cells exposed to ArA (Table 2, *p*-value: 0.0016) and BAD downregulated in cells exposed to OvC3 and ArA (Table 2, *p*-value: < 0.001). Overexpression of CRLF3 has been shown to cause G0/G1 arrest and prevent S phase entry in human embryo kidney 293T cells [55]. Moreover, Rodolosse et al. showed that CRLF3 resulted in increased levels of hypophosphorylated retinoblastoma (Rb) protein through presumably upregulation of cyclin-dependent kinase (Cdk) inhibitors (i.e. p21^CIP1/WAF1^ and p27^KIP1^) and downregulation of cyclin D2. In this form, the Rb protein binds and thereby inactivates the E2 factor resulting in the suppression of genes necessary for G1 phase progression [56]. Previous studies reported a cell cycle regulating effect of TGF-β and IL-6 [57,58]. TGF-β has been shown to restrict G1 phase progression in epithelial cells through the suppression and stimulation of c-myc and Cdk inhibitors transcription, respectively [59]. Presumably, inhibition of c-myc and activation of Cdk inhibitors results in the inhibition of Cdk2/Cdk4 preventing phosphorylation of the Rb protein and delaying G1 phase progression [60–62]. IL-6 was reported to induce both cell growth inhibitory and stimulatory signals [63]. In hepatocellular carcinoma cells IL-6 exposure resulted in the induction of Cdk inhibitor (i.e. p21^CIP1/WAF1^) through the Janus kinase and signal transducer and activator of transcription protein 3 (JAK-STAT3) signaling pathway [58]. Moreover, an upregulation of phosphorylated STAT3 was observed after overexpression of CRLF3 [55]. Both, TGF-β and IL-6 were shown to delay G1 phase progression through signaling pathways in which CRLF3 seems to be involved. However, the precise mechanism is not conclusively resolved.

CCNB2 in complex with Cdk1 has been shown to be involved in the process of centrosome separation during G2 phase [64]. CCNB2 depletion significantly reduced the activation of downstream proteins and increased the number of asymmetrical mitotic spindles and chromosome lagging [64]. DNA damage, but also exposure to TGF-β, were shown to inactivate the CCNB2/Cdk1 complex leading to cell cycle arrest by preventing the entry into G2/M phase [65,66]. However, Nam et al. showed recently that the depletion of CCNB2 in SV40 large T antigen immortalized mouse fibroblasts failed to inhibit the centrosome separation due to abnormal function of the CCNB2 antagonist and tumor suppressor gene p53. Moreover, loss of p53 was accompanied with CCNB2-independent activation of downstream proteins [64]. In this study, only MeT-5A cells exposed to patient-derived ascites showed a downregulation of CCNB2 excluding TGF-β as a potential stimulus. We presume, therefore, that another factor of patient-derived ascites is responsible for the downregulation of CCNB2. Moreover, it remains open if a significant effect on the activity of downstream proteins upon CCNB2 downregulation can be expected in the SV40 large T antigen immortalized MeT-5A cells.

The shelterin protein TRF2 provides the formation of a telomeric t-loop to protect the integrity of the telomeres and prevent chromosomal end-to-end fusion [67]. An upregulation of TRF2 was shown to act as an anti-apoptotic mechanism in malignant cells in response to DNA damage [68]. Moreover, it was recently described that TRF2 inhibits the completion of DNA double-strand break repair in the telomeric DNA region leading to cell cycle blockade through the ataxia telangiectasia mutated (ATM) signaling to p53 and/or p16 [69,70]. The phosphorylation of p53 through ATM has been shown to express pro-survival genes presumably as early response required for cell cycle checkpoint activation and DNA repair [69]. Furthermore, ATM results in the activation of the protein kinase B (AKT), which was shown to suppress apoptosis by inhibiting pro-apoptotic proteins including BAD [71,72]. The phosphoinositide 3-kinase (PI3K)/AKT signaling pathway was also shown to be involved in the TGF-β induced EMT through NF-κB/Snail signaling. Activation of the NF-κB/Snail signaling pathway leads in EMT phase I to disruption of intercellular adhesion and loss of polarity and in EMT phase II to cell growth arrest and cell survival [13]. We presume that exposure to high TGF-β1 concentrations as it was the case mainly for ArA might have induced a shift to phase II EMT in MeT-5A cells. To further evaluate a possible reduced cell proliferation, the number of MeT-5A cell nuclei per condition (nine images, three per repetition) was assessed. The sum (and median) of the three repetitions were 242 (73), 253 (85), 265 (86), and 247 (86) nuclei for OvC3, OvC4, PeC, and ArA, respectively, which is lower than the sum of untreated cells with 270 (98) nuclei. We, therefore, assume that exposure to mainly ArA might have shifted MeT-5A cells into phase II EMT including lower cell proliferation and activated pro-survival pathways.

### Untreated MeT-5A cells show reduced cell-cell interactions

**Fig 2:**
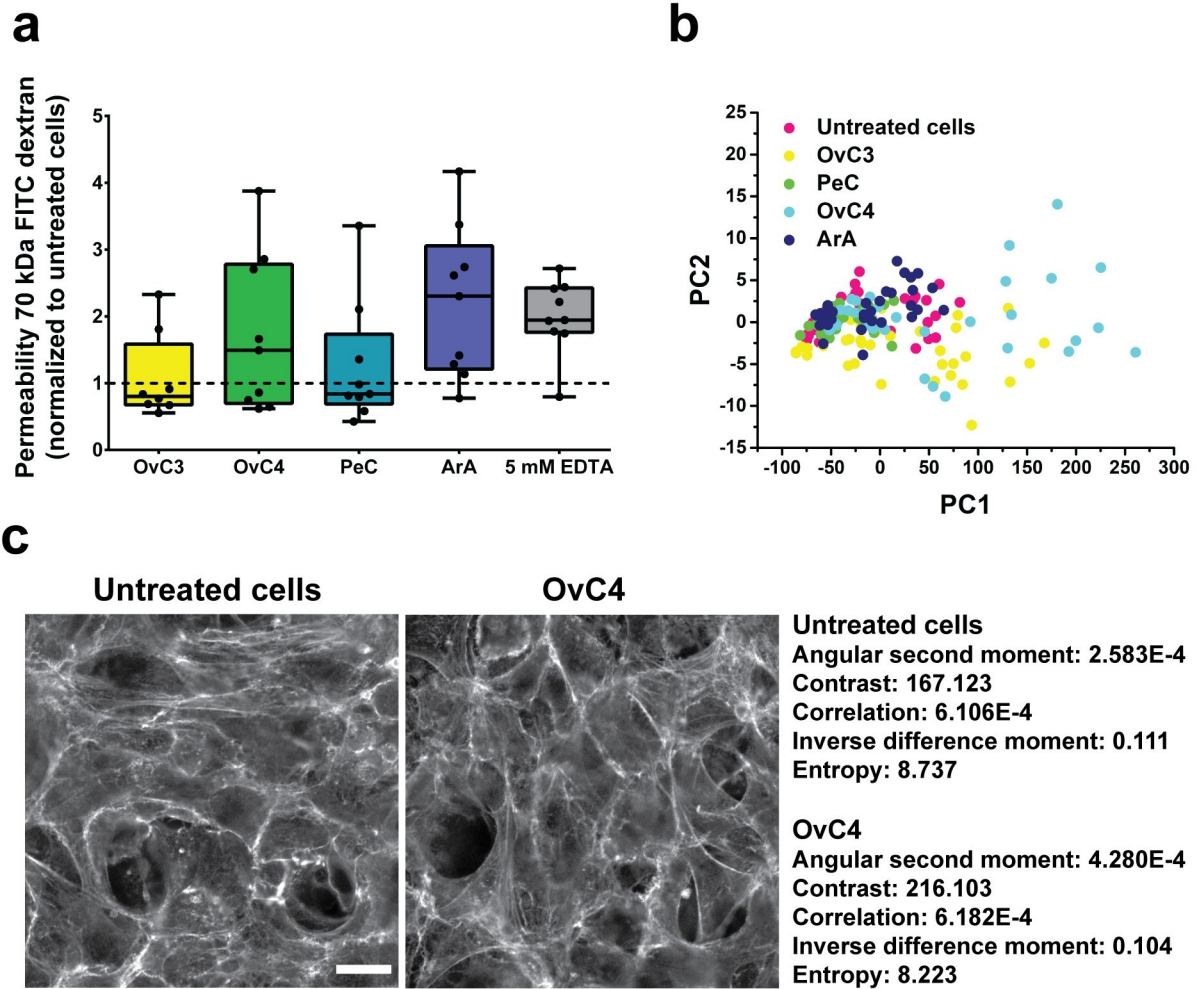
Permeability and F-actin texture analysis of MeT-5A cells. a) Permeability of exposed and EDTA treated (positive control) cells normalized to untreated cells (dotted line). Data are presented as median ± Min/Max, n = 3. b) PCA of F-actin texture showing the first two PCs with the highest variance. c) Example of GLCM of untreated and OvC4 treated MeT-5A cells. Scale bar is 20 μm.

Changes in cell-cell interactions of MeT-5A cells exposed to ascites were investigated using a FITC-dextran permeability assay. A non-significant 1.49- and 2.3-fold increase in cell layer permeability was determined for cells exposed to OvC4 and ArA, respectively, whereas a slightly increased barrier integrity was measured for OvC3 and PeC (0.8- and 0.84-fold, Kruskal-Wallis test, Fig 2A). Exposure to the positive control EDTA resulted in increased permeability, which was close to be statistically significant when compared to untreated cells (unpaired *t*-test, *p*-value: 0.0557). As the increase in paracellular permeability is mainly caused by disrupted tight junctions (TJ) [73], the expression level of the TJ proteins zonula occludens-1 and -2 (ZO-1 and ZO-2) in MeT-5A cells exposed to ascites was analyzed. No statistically significant downregulation of the two proteins was observed in exposed compared to untreated cells (Table 2). Moreover, the immunofluorescence staining of the TJ proteins ZO-1 and occludin in untreated MeT-5A cells revealed only unspecific TJ staining, indicating no formation of mature TJ between neighboring cells (S2 Fig). These results might indicate that untreated cells show already reduced cell-cell interactions, which may explain the less pronounced effect of the positive control EDTA. Interestingly, Aguilera et al. observed that mesothelial cells isolated from the peritoneal dialysis effluent showed a transitional EMT phenotype. This transitional phenotype shows, among others, a downregulation of E-cadherin when compared to cobblestone mesothelial cells [13]. The MeT-5A cells origin from the pleural fluid and strongly resemble the morphology of transitional mesothelial cells (S3 Fig). Moreover, EGF, which is a supplement of the complete cell culture medium was shown to induce EMT [5]. Song et al. previously demonstrated that exposure to TGF-β1 decreased ZO-1 and E-cadherin protein content in MeT-5A cells cultured in medium without EGF [43]. Therefore, we presume that untreated MeT-5A cells display a transitional phenotype with phase I EMT including impaired cell-cell interactions and loss of cell polarity. Nevertheless, cells exposed to ArA showed a trend towards higher permeability, which might be due to cell retraction. Cytoskeleton rearrangement including downregulation of stress fiber formation is typical for EMT phase III and was shown to be induced by exposure to proinflammatory environment [13,17].

### Ascites did not induce cytoskeleton rearrangement

The results of the permeability study indicated a possible cell retraction in cells exposed to ArA. To analyze cytoskeleton rearrangement including reduced stress fiber formation in cells, PCA of F-actin texture (GLCM, Fig 2C), intensity, and number of nuclei was evaluated. This analysis was chosen as F-actin staining of ascites-exposed pHPMC revealed significant reduction in stress fibers formation and increased intercellular gap area [17]. Additionally, the expression of CDC42 effector protein 2 (CDC42EP2), which was shown to be involved in the reduction of F-actin stress fiber thickness and organization [74] and α-smooth muscle actin (ACTA2), a marker of phase III EMT, were analyzed. In PCA analysis, the first two resulting PCs with the highest variance were plotted for visual comparison of the separation/classification between every sample (Fig 2B). The 2D scatter plot of PC1 and PC2 revealed no differences in the spatial distribution of all experimental data sets, indicating that each image featured similar data. The sample-to-sample variation within each condition was probably higher than the differences between the different conditions, which leveled out possible changes. The protein expression analysis of CDC42EP2 showed a statistically significant upregulation for cells treated with OvC3 and ArA (Table 2, empirical Bayes moderated *t*-tests, *p*-value: 0.0095 (OvC3) and < 0.0001 (ArA)). ACTA2, however, was significantly downregulated for cells exposed to ArA (Table 2, empirical Bayes moderated *t*-tests, *p*-value: 0.0095). Compared with reported studies about cell retraction in pHPMC *in vitro*, PeC and ArA showed sufficiently high cytokine concentrations to induce cytoskeleton rearrangement (S1 Fig). We presume that exposure to mainly ArA initiated stress fiber reorganization as illustrated in upregulated CDC42EP2 expression. However, EMT phase III including cytoskeleton rearrangement and upregulation of ACTA2 was not observed. Moreover, sample-to-sample variation was presumably too high to detect differences between conditions using PCA.

### Fibronectin production is not affected by exposure to ascites

FN1 is a key ECM protein providing integrin-mediated cell adhesion and intracellular signal transduction, and was shown to be upregulated in mesothelial cells with fibroblastic phenotype [13,18]. Moreover, increased FN1 expression was observed in ovarian cancer metastases and induced ovarian cancer cell adhesion, proliferation, and invasion [18]. Intracellular FN1 expression in MeT-5A cells exposed to ascites was determined using proteomics. Protein expression did not show any statistically significant upregulation (Table 2, empirical Bayes moderated *t*-tests), although high TGF-β1 concentrations were measured in all ascites (Table 1, S1 Fig). As untreated MeT-5A cells show probably a transitional EMT phenotype including upregulated FN1 production, it can be assumed that exposure to TGF-β1 or IL-1β did not promote a significant FN1 upregulation in cells exposed to ascites [5,13,14].

### Minor cell surface changes in MeT-5A cells exposed to artificial and patient-derived ascites

Surface changes of MeT-5A cells were investigated by analyzing ICAM-1 and VCAM-1 expression, HA secretion, and microvillus expression. Cells exposed to ArA showed a small but statistically significant increase in ICAM-1 expression (empirical Bayes moderated *t*-tests, *p*-value: 0.003), while no effect was observed for the other conditions and VCAM-1 expression (Table 2). According to the cytokine concentration (Table 1, S1 Fig), ArA showed sufficiently high concentrations to significantly upregulate ICAM-1 and VCAM-1 expression, while PeC could have theoretically induced upregulation of ICAM-1. Previous studies have shown that EGF induces EMT, leading to a more fibroblastic phenotype in pHPMC [75]. Interestingly, Yuan et al. demonstrated recently that EGF exposure to primary pleural mesothelial cells derived from tuberculous pleural effusions (pPMC TPE) did not affect ICAM-1 and VCAM-1 expression. Moreover, exposure to TGF-β and interleukin-4 (IL-4), which induced a significant change in pPMC TPE, revealed unchanged ICAM-1 and VCAM-1 expression in MeT-5A cells. Only interferon-γ resulted in a significant upregulation of ICAM-1 expression in MeT-5A cells, however, the increase observed was lower than in pPMC TPE. VCAM-1 expression remained unaffected in MeT-5A cells upon stimulation [76]. This study validates the assumption that MeT-5A cells show a reduced biological responsiveness to proinflammatory stimuli than primary cells. ICAM-1 and VCAM-1 are cell-adhesion proteins, which usually serve as binding sites for leukocytes. Proinflammatory cytokines (IL-6, IL-1β or TNF-α) were shown to upregulate ICAM-1 and VCAM-1 expression to provide increased binding sites for adhesion [20,22]. However, cancer cells also show an increased integrin-mediated adhesion to mesothelial cells when ICAM-1 and VCAM-1 were upregulated [22,29,30].

**Fig 3:**
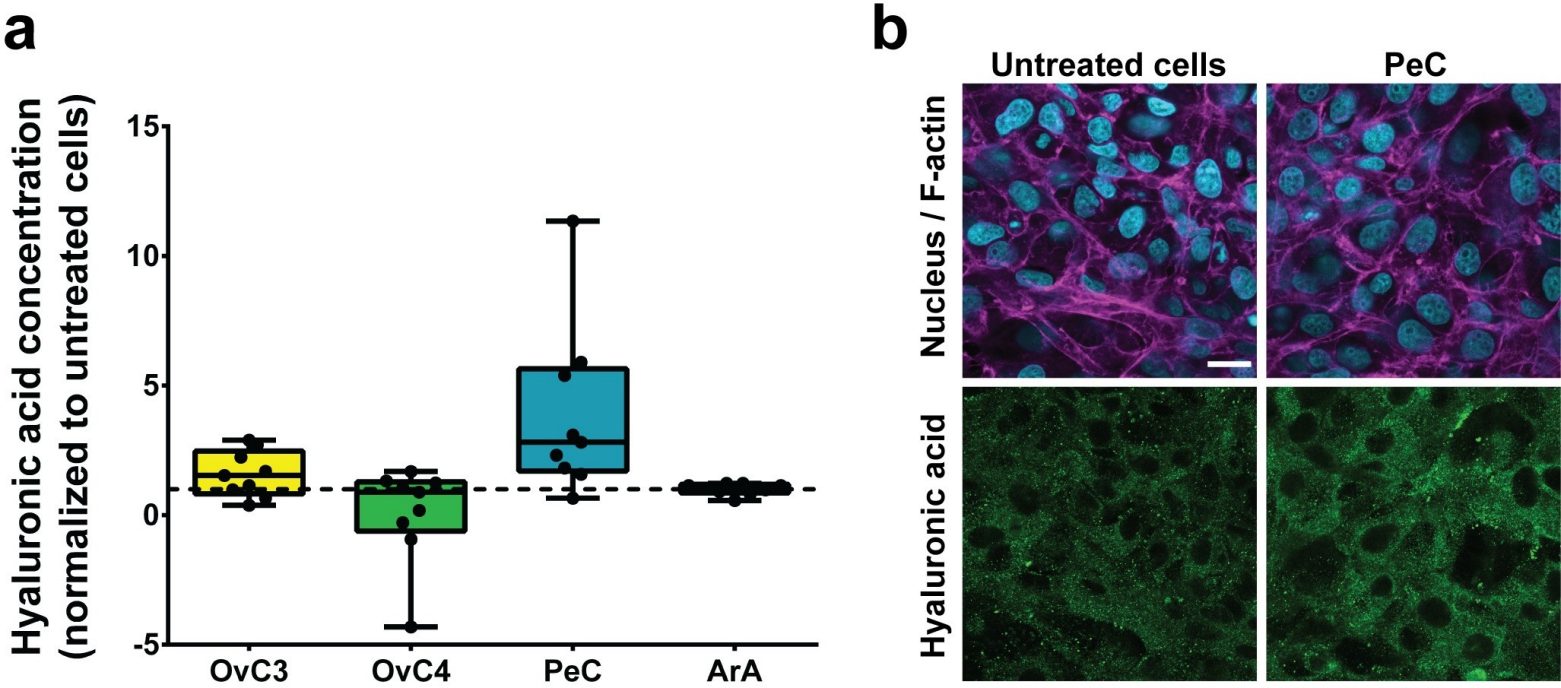
Hyaluronic acid concentration of exposed MeT-5A cells compared to untreated cells. a) HA secretion of cells exposed to ascites normalized to untreated samples (dotted line); data are presented as median ± Min/Max, n = 3. b) Fluorescence images of untreated MeT-5A cells and exposed to PeC. Cell nuclei are displayed in cyan, F-actin in magenta, and HA in green. Scale bar is 20 μm.

In addition, the HA-rich surface of mesothelial cells allows the binding of ovarian cancer cells via the HA receptor CD44 [11]. To evaluate if exposure to ascites increased HA secretion of MeT-5A cells, HA concentration of the supernatant was analyzed using ELISA. Moreover, the presence of HA within the cells was visualized using LSM. HA concentration was increased when cells were exposed to OvC3 and PeC (1.53- and 2.81-fold, respectively), slightly decreased when exposed to OvC4 (0.89-fold), and remained unchanged for ArA (1.05-fold, Kruskal-Wallis test, *p*-value > 0.05, Fig 3A). The cytokine profiling of ascites presupposed a change in HA secretion by MeT-5A cells exposed to OvC3, PeC, and ArA (Table 1, S1 Fig). However, only a trend towards higher HA secretion was observed in cells exposed to PeC. Yung et al. showed that pHPMC exposed to FBS were conditioned to synthesize HA [24]. We assume that stimulation with the cell culture medium supplement FBS, might have pre-stimulated cells to secrete HA and, therefore, an additional stimulation with proinflammatory cytokines did not enhance significantly HA secretion.

**Fig 4:**
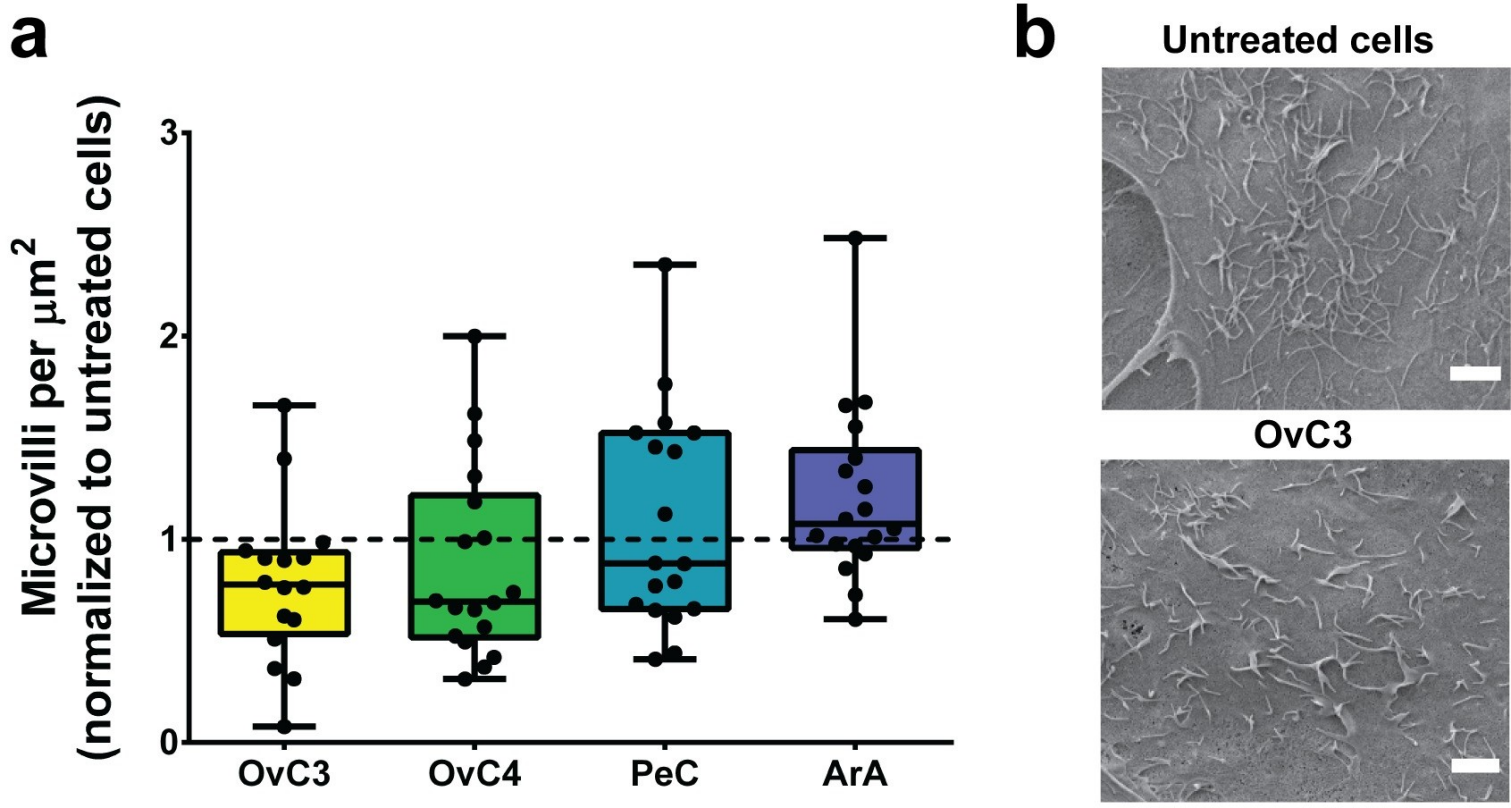
Microvillus expression of MeT-5A cells exposed to ascites compared to untreated cells. a) Number of microvilli of cells exposed to ascites normalized to untreated cells (dotted line); data are presented as median ± Min/Max, n = 3. b) Exemplary SEM images of microvilli, scale bars are 2 μm.

To visualize the presence of HA within the cells, HA was fluorescently stained and analyzed using LSM. Vesicle-like structures distributed throughout the entire cell were observed (Fig 3B). Secreted HA is trapped between the microvilli expressed on the apical side of the mesothelial cells to protect from abrasive damage [77]. Lower numbers of expressed microvilli were observed in mesothelial cells exposed to ascites *in vivo* [16]. Therefore, the number of microvilli (Fig 4A) was evaluated in MeT-5A cells exposed to patient-derived and artificial ascites. Analysis of SEM images (exemplary images are shown in Fig 4B) revealed that cells exposed to OvC3, OvC4, and PeC showed fewer microvilli on the cell surface than untreated cells (0.78-, 0.69-, and 0.88-fold), however, differences were not statistically significant (Kruskal-Wallis test). Cells exposed to ArA showed an equal number of microvilli as untreated cells (1.08-fold, Fig 4A). As the core of the microvilli consists of a bundle of F-actin, this observation might indicate that the F-actin organization involved in microvilli formation remained unaffected.

### Single-cell adhesion of SKOV-3 on MeT-5A cells is not facilitated by ascites

**Fig 5:**
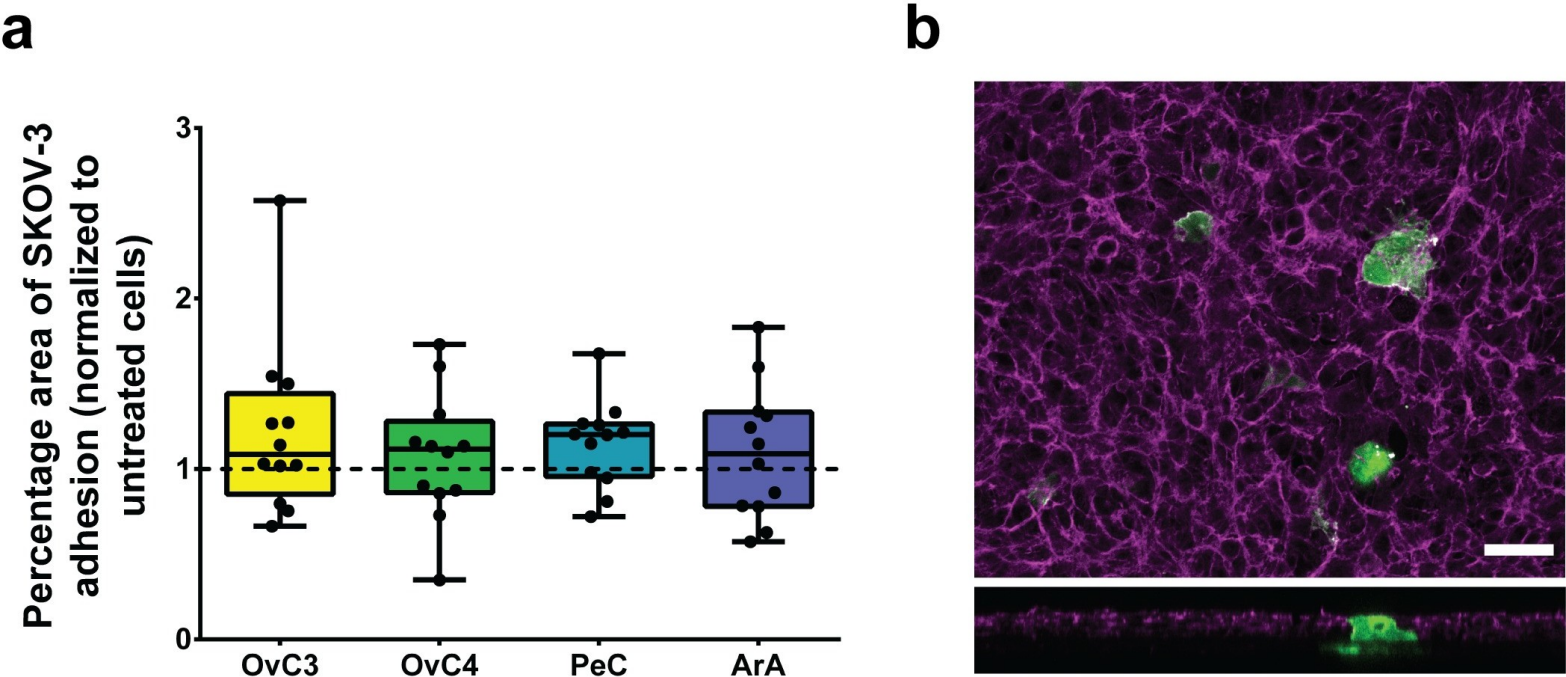
SKOV-3 cell adhesion on exposed MeT-5A cells compared to untreated cells. a) Percentage area of SKOV-3 cell adhesion on mesothelial cells when normalized to untreated cells (dotted line); data are presented as median ± Min/Max, n = 2. b) SKOV-3 cell (green) adhesion on and through the MeT-5A cell layer (magenta, exposed to OvC4), scale bar is 50 μm.

Exposure to a proinflammatory environment leads to mesothelial cell retraction, upregulated ICAM-1 or VCAM-1 expression, and increased HA secretion and FN1 production, which have been shown to facilitate ovarian cancer cell adhesion both *in vitro* and *in vivo* [11,17,18,22,26–29]. To investigate if the morphological and functional changes mainly observed in MeT-5A cells exposed to ArA would allow an increased SKOV-3 cell adhesion to the MeT-5A cells and the underlying collagen layer, the percentage area of SKOV-3 cells on exposed MeT-5A cell layers was evaluated and compared to untreated cells. The cell line SKOV-3 was chosen as they were extracted from ascites of a patient with an adenocarcinoma of the ovary representing the *in vivo* situation. After 24 h, SKOV-3 cell adhesion on MeT-5A cells was only slightly increased relative to untreated cells and showed values of 1.09-, 1.12-, 1.2-, and 1.09-fold for OvC3, OvC4, PeC, and ArA, respectively (Figs 5A and S4, Kruskal-Wallis test, *p*-value > 0.05). These findings allow us to assume that the observed morphological and functional changes in MeT-5A cells exposed to ascites were not sufficient to significantly affect SKOV-3 cell adhesion. However, we observed that SKOV-3 cells were able to penetrate the MeT-5A cell layer, presumably using mesothelial cell clearance as previously suggested by Iwanicki et al. [27] (Fig 5B). Nevertheless, previous studies indicated that proinflammatory cytokines facilitate cancer cell adhesion on mesothelial cells [17,78]. Van Rossen et al. showed that exposure of IL-1β and TGF-β to rat primary mesothelial cells led to increased colon carcinoma cell adhesion of 60% and 16%, respectively. However, IL-6 and TNF-α did not significantly change cancer cell adhesion [78]. Contrary to these results, a study with pHPMC showed a 3-fold higher gastric cancer cell adhesion when exposed to TNF-α [17]. However, our study did not suggest an increased SKOV-3 cell adhesion on MeT-5A cells exposed to ascites. We presume that untreated MeT-5A cells show a transitional EMT rather than cobblestone epithelioid phenotype lacking functional cell-cell interactions and upregulating FN1 production [14,75]. Additionally, HA secretion might have already been stimulated with FBS [24] or the HA layer could have been disintegrated, leading to soluble HA, which interacted with the CD44 antigens of ovarian cancer cells [11]. These factors could have limited the number of binding sites and SKOV-3 cell adhesion on MeT-5A cells was not facilitated.

## Conclusion

The herein presented study provides experimental evidence that MeT-5A cells are less responsive when exposed to ascites than previously reported for primary human peritoneal mesothelial cells and thus do not serve as an optimal system to study cancer cell adhesion and migration. The lower biological responsiveness of MeT-5A cells may result from (i) lower sensitivity of cell lines compared to primary cells as observed in ICAM-1/VCAM-1 expression, (ii) transitional EMT phenotype of untreated cells, and (iii) pre-stimulation of untreated cells with supplements (i.e. FBS and EGF) of the complete cell culture medium. We hypothesize that untreated MeT-5A cells show a phase I EMT phenotype including reduced cell-cell interactions and loss of cell polarity. Nevertheless, exposure to high cytokine concentrations in ArA induced cell cycle arrest and activated survival pathways presumably shifting MeT-5A cells to phase II EMT. Moreover, exposure to ArA affected MeT-5A cell morphology and functionality to a higher extent than patient-derived ascites and an anti-inflammatory response was observed presumably protecting cells from cytokine-induced cell damage. This observation may be an indication that higher cytokine concentrations or longer incubation times are required to significantly induce effects in MeT-5A cells.

## Supporting information

S1 Table Pathological information of ascites donors. (PDF)

S1 File Detailed protocol for proteomics. (PDF)

S1 Fig Cytokine concentration of patient-derived ascites and limit concentrations required to induce effects in primary mesothelial cells in *in vitro*. Cytokine concentration of ascites is compared to limit concentrations required to induce effects on a) cell retraction, b) ICAM-1, c) VCAM-1, d) HA, and e) FN1. Darkened bars represent cytokine concentrations that reach the concentrations required to induce an effect. Dotted lines show limit concentrations of different cytokines. Effects of 4 times diluted concentrations to consider a possible diffusion through the cell layer were evaluated additionally.
(TIF)

S2 Fig Tight junction staining of untreated MeT-5A cells. MeT-5A cells were cultured for 5 days and immuno-fluorescently stained using a) ZO-1 and b) occludin. Scale bar is 20 μm.
(TIF)

S3 Fig Light microscope images of untreated MeT-5A cells. Confluent cell layer shown at two different magnifications. Scale bar is in a) 200 μm and b) 100 μm.
(TIF)

S4 Fig SKOV-3 cells adhesion. Representative images of SKOV-3 cells on the MeT-5A cell layer (color in images not shown). Scale bar is 50 μm.
(TIF)
