# Supplementary material for: Patient-derived and artificial ascites have minor effects on MeT-5A mesothelial cells and do not facilitate ovarian cancer cell adhesion
Source: PLoS One. 2020 Dec 3;15(12):e0241500. doi: 10.1371/journal.pone.0241500 (PMC7714103; doi:10.1371/journal.pone.0241500)
Supplement: S1 Table — (PDF) [file pone.0241500.s001.pdf]

S1 Table. Pathological information of ascites donors.

Patient	Age	Diagnosis	Histotype	Grade	FIGO stage	Ascites collection	Malignant cells	CA125 [kU/l]
OvC3	71	Ovarian cancer	serous	3	III	at primary diagnosis	yes	560
OvC4	78	Ovarian cancer	serous	3	IV	at primary diagnosis	yes	180
PeC	58	Peritoneal cancer	serous	3	IV	at primary diagnosis	yes	703
