# Supplementary material for: Patient-derived and artificial ascites have minor effects on MeT-5A mesothelial cells and do not facilitate ovarian cancer cell adhesion
Source: PLoS One. 2020 Dec 3;15(12):e0241500. doi: 10.1371/journal.pone.0241500 (PMC7714103; doi:10.1371/journal.pone.0241500)
Supplement: S1 File — (PDF) [file pone.0241500.s002.pdf]

S1 File. Detailed protocol for proteomics

Lysis buffer containing 1 % sodium deoxycholate (SDC, 89904, Thermo Scientific Scientific, Switzerland), 10 mM tris(2-carboxyethyl)phosphine (TCEP, 646547, Sigma-Aldrich, Switzerland) in 100 mM tris(hydroxymethyl)aminomethane (Tris, pH 8.5) was added to cells and samples were sonicated using the Bioruptor (Diagenode, Belgium) with 10 cycles (30 sec on and 30 sec off). Next, the samples were incubated at 95 °C for 10 min at 300 rpm. The protein concentration of the samples was measured using the standard bicinchoninic acid (BCA) assay according to the manufacturer's instruction (23225, Thermo Fisher Scientific, Switzerland). 50 µg of total protein lysates was alkylated with 1 µL of 0.75 M chloroacetamide for 30 min at 37 °C (500 rpm), and digested with sequencing-grade modified trypsin (1/50, w/w; Promega, Madison, Wisconsin) overnight at 37 °C. Digested peptides were cleaned using in-StageTip (iST) cartridges (PreOmics, Munich, Germany) according to the manufacturer's instruction. Samples were resuspended in 0.1 % formic acid by sonication and subjected to LC-MS/MS analysis using an Orbitrap Fusion Lumos Tribrid Mass Spectrometer fitted with an EASY-nLC 1200 (Thermo Fisher Scientific, Switzerland) and a custom-made column heater set to 60 °C. Peptides were resolved using an RP-HPLC column (75 µm × 36 cm) and packed in-house with C18 resin (ReproSil-Pur C18–AQ, 1.9 µm resin; Dr. Maisch GmbH, Germany) at a flow rate of 200 nL/min. The following gradient was used for peptide separation: from 5 % B to 12 % B (5 min), to 35 % B (65 min), to 50 % B (20 min), to 95 % B (2 min) followed by 18 min at 95% B. Buffer A was 0.1 % formic acid in water and buffer B was 80 % acetonitrile, 0.1 % formic acid in water.

The mass spectrometer was operated in DDA mode with a cycle time of 3 seconds between each MS1 scans. Each MS1 scan was acquired in the Orbitrap at a resolution of 240'000 full width at half maximum (FWHM, 200 m/z) and a scan range from 375 to 1600 m/z. This was followed by MS2 scans of the most intense precursors in the linear ion trap at 'Rapid' scan rate with isolation of the quadrupole set to 1.4 m/z. Maximum ion injection time was set to 50 ms (MS1) and 35 ms (MS2) with an AGC target set to '250 %' and 'Standard', respectively. Only

peptides with charge states between 2 – 5 were included in the analysis. Monoisotopic precursor selection (MIPS) was set to ‘Peptide’, and the ‘Intensity Threshold’ was set to 5e3. Peptides were fragmented by higher-energy collisional dissociation (HCD) with collision energy set to 35 %. One microscan was acquired for each spectrum. The dynamic exclusion duration was set to 30 s.

The acquired raw-files were imported into the Progenesis QI software (v2.0, Nonlinear Dynamics Limited, United Kingdom), which was used to extract peptide precursor ion intensities across all samples applying the default parameters. The generated mgf-file was searched using MASCOT against a human database containing 20’416 protein sequences downloaded from Uniprot on 20190129 using the following search criteria: full tryptic specificity was required (cleavage after lysine or arginine residues, unless followed by proline); 3 missed cleavages were allowed; carbamidomethylation (C) was set as fixed modification; oxidation (M) and acetyl (protein N-term) were applied as variable modifications; mass tolerance of 10 ppm (precursor) and 0.6 Da (fragments). The database search results were filtered using the ion score to set the false discovery rate (FDR) on the peptide and protein level to 1 % based on the number of reverse protein sequence hits in the dataset. The relative quantitative data obtained were normalized and statistically analyzed using the in-house script ‘Safe Quant’ (PMID:27345528). Quantitative analysis results from label-free quantification were processed using the SafeQuant R package v.2.3.2. (https://github.com/eahrne/SafeQuant/) to obtain peptide relative abundances. This analysis included global data normalization by equalizing the total peak/reporter areas across all LC-MS runs, data imputation using the knn algorithm, the summation of peak areas per protein and LC-MS/MS run, followed by calculation of peptide abundance ratios. Only isoform-specific peptide ion signals were considered for quantification. The summarized peptide expression values were used for statistical testing of differentially abundant peptides between conditions. Here, the empirical Bayes moderated *t*-tests were applied, as implemented in the R/Bioconductor limma package (http://bioconductor.org/packages/release/bioc/html/limma.html).
