# Supplementary figures and images for: Patient-derived and artificial ascites have minor effects on MeT-5A mesothelial cells and do not facilitate ovarian cancer cell adhesion
Source: PLoS One. 2020 Dec 3;15(12):e0241500. doi: 10.1371/journal.pone.0241500 (PMC7714103; doi:10.1371/journal.pone.0241500)

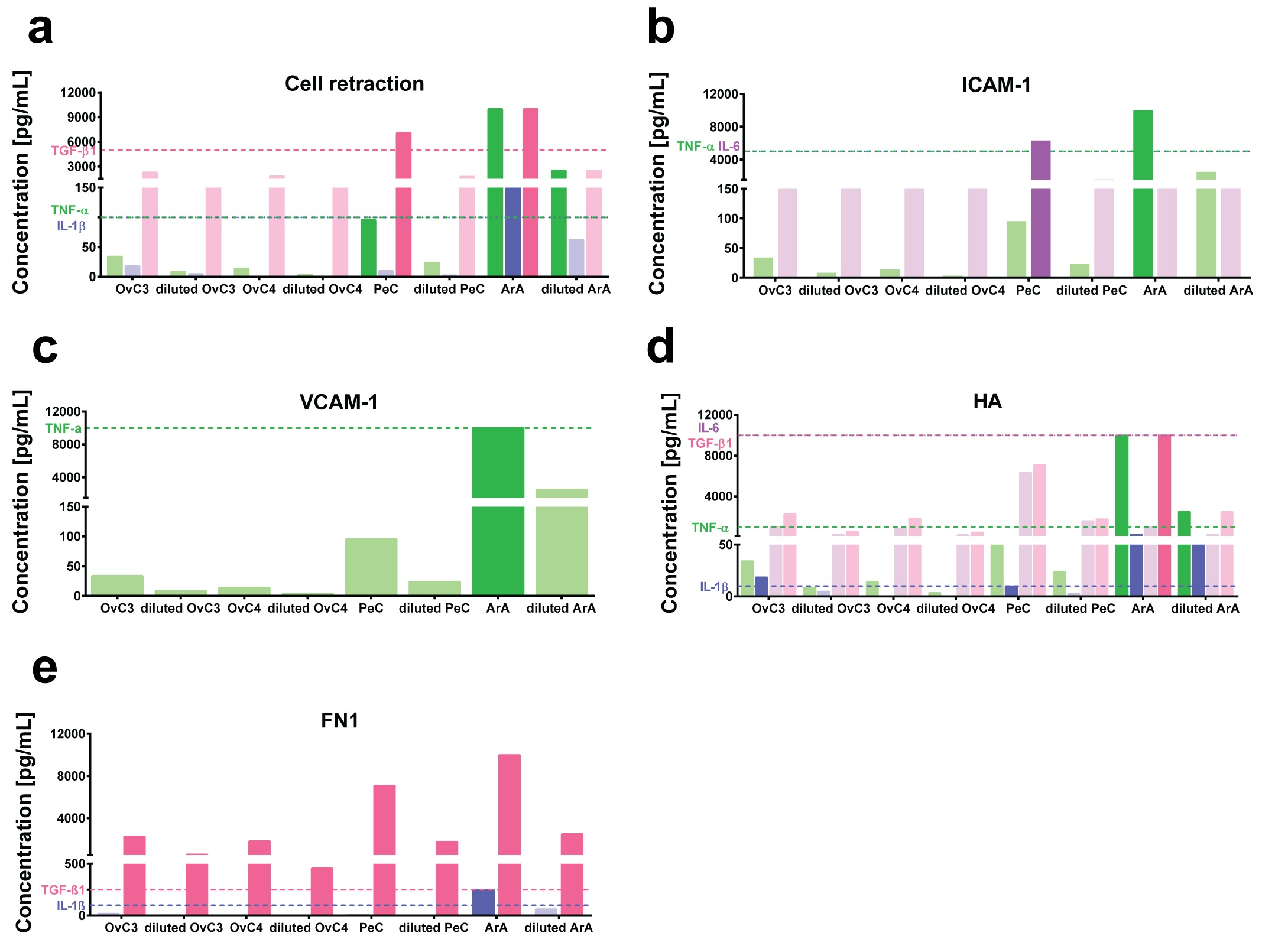

Supplement: S1 Fig — Cytokine concentration of ascites is compared to limit concentrations required to induce effects on a) cell retraction, b) ICAM-1, c) VCAM-1, d) HA, and e) FN1. Darkened bars represent cytokine concentrations that reach the concentrations required to induce an effect. Dotted lines show limit concentrations of different cytokines. Effects of 4 times diluted concentrations to consider a possible diffusion through the cell layer were evaluated additionally. (TIF) [file pone.0241500.s003.tif]

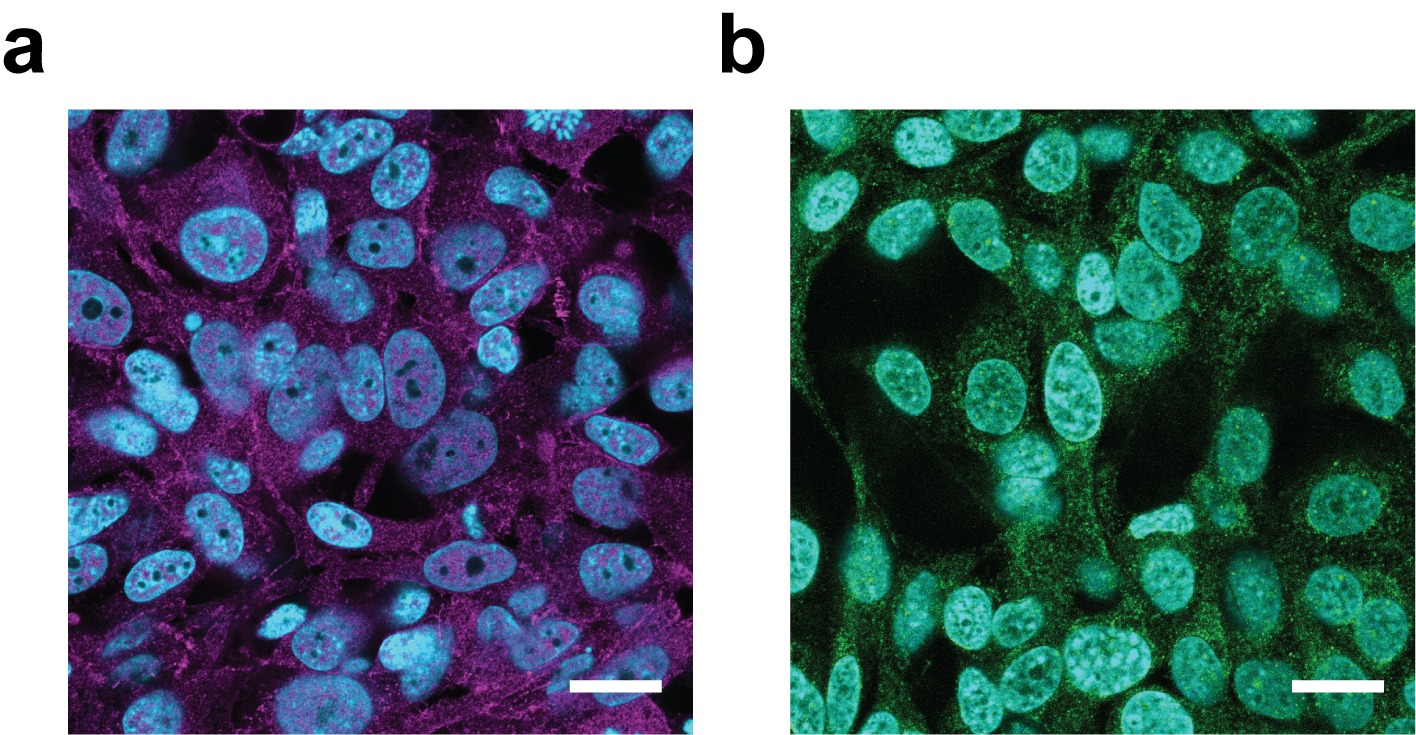

Supplement: S2 Fig — MeT-5A cells were cultured for 5 days and immuno-fluorescently stained using a) ZO-1 and b) occludin. Scale bar is 20 μm. (TIF) [file pone.0241500.s004.tif]

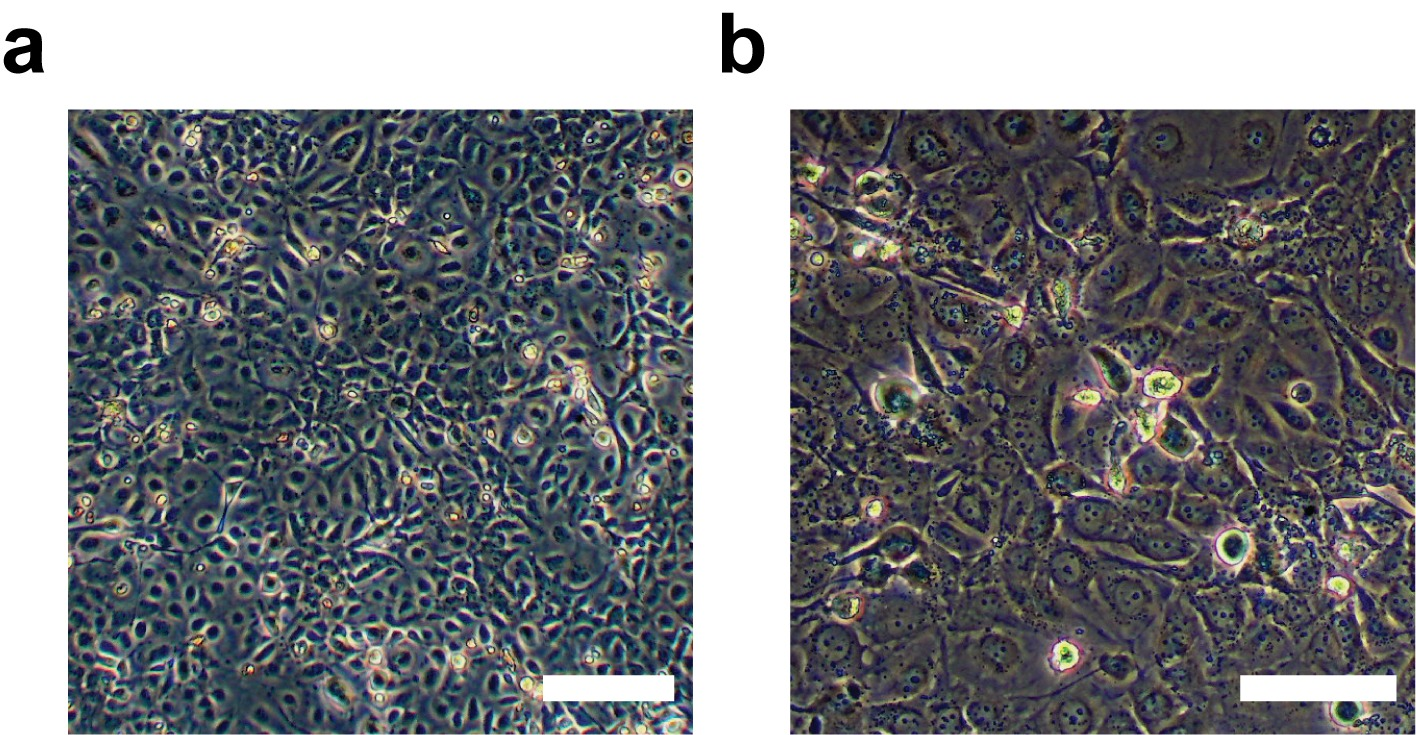

Supplement: S3 Fig — Confluent cell layer shown at two different magnifications. Scale bar is in a) 200 μm and b) 100 μm. (TIF) [file pone.0241500.s005.tif]

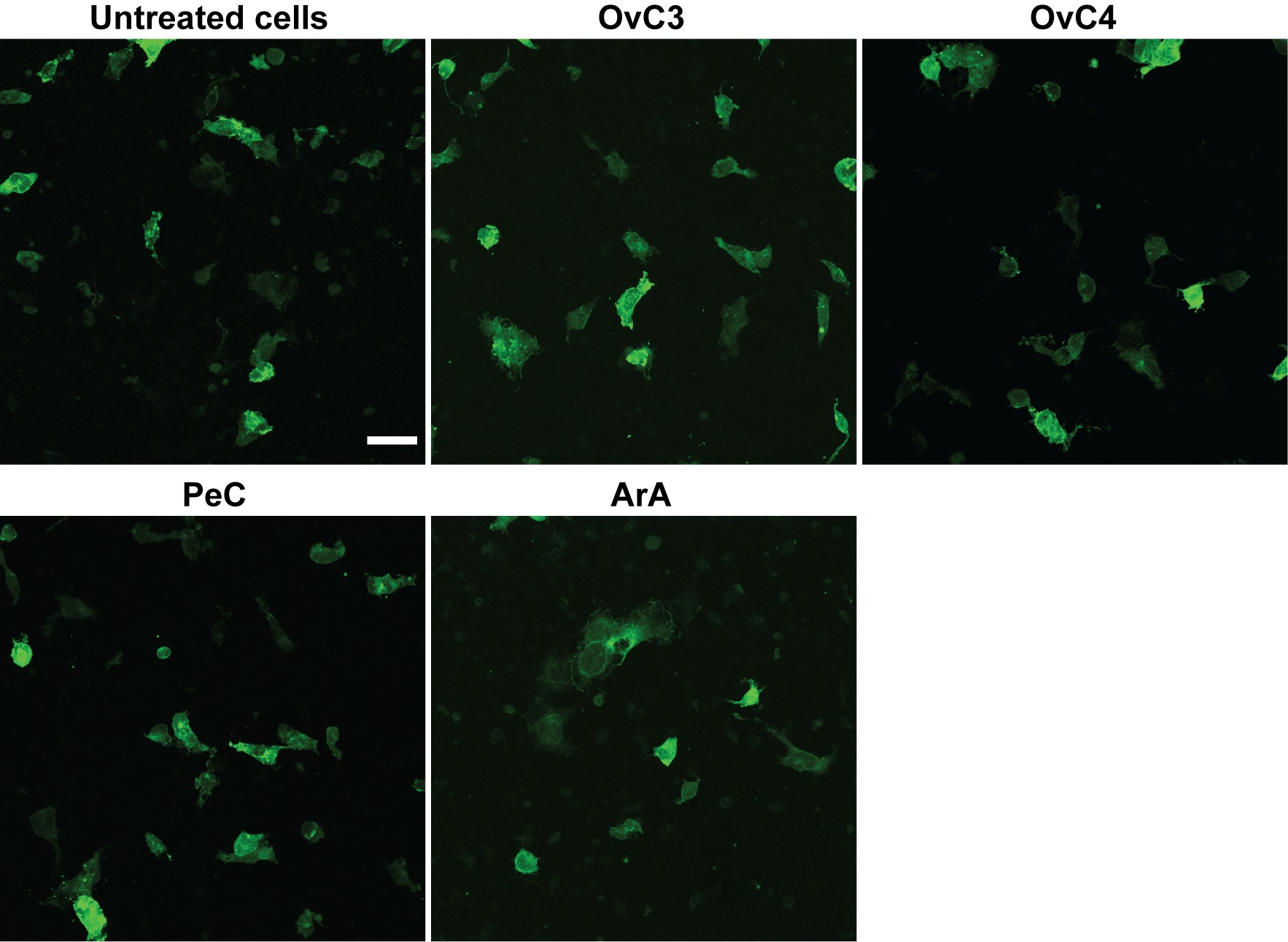

Supplement: S4 Fig — Representative images of SKOV-3 cells on the MeT-5A cell layer (color in images not shown). Scale bar is 50 μm. (TIF) [file pone.0241500.s006.tif]
